# Electrocutions in Free‐Ranging Platyrrhine Nonhuman Primates: Diagnostic Features for a Threatening Condition

**DOI:** 10.1002/ajp.70039

**Published:** 2025-04-20

**Authors:** Rafaela M. Barros, Isabel L. Macêdo, Davi E. R. Sousa, Liz A. Cerqueira, Yasmin N. G. Fonseca, Ana L. V. Sousa, Antonio D. Santos, Cristiano B. de Melo, Márcio B. Castro

**Affiliations:** ^1^ Graduate Program in Animal Science University of Brasília Brasilia Brazil; ^2^ Veterinary Pathology and Forensics Laboratory University of Brasilia Federal District Brazil

**Keywords:** conservation, electric burns, marmoset, skin metallization, thermic injury

## Abstract

Electrical accidents pose a considerable threat to wildlife in anthropized regions, particularly arboreal mammals, as evidenced by cases of electrocutions in nonhuman primates (NHPs). This study characterized the frequency, anatomic distribution, and pathological features of electrocution injuries in free‐ranging NHPs based on data retrieved from necropsy archives at the Veterinary Pathology and Forensics Laboratory, University of Brasilia, Brazil. Gross and microscopic evaluations were conducted to assess the nature and extent of electrothermal injuries, including burns and tissue damage. Between 2019 and 2022, electrocution accounted for 16.5% of NHP deaths, primarily affecting black tufted marmosets. Adult NHPs, regardless of sex, were predominantly affected, mainly in the rainy season. Limbs were the most commonly affected body parts, with double, triple, or multiple injury sites being common. Gross findings mostly included severe burns (Grades III and IV), ulcerative injuries, blisters, bone exposure, singed hair, and muscle exposure. Microscopic examinations unveiled epidermal necrosis, smudging of collagen fibers, stretched epidermal nuclei, blisters (epidermal honeycomb aspect), muscle necrosis, skin metallization, and hemorrhage and congestion within internal organs. Electrocution has emerged as a substantial cause of death in free‐ranging NHPs, particularly in urban and peri‐urban areas. The study underscores the need for public policy adjustments to mitigate the risks of electrocution in NHPs and enhance species conservation efforts in human‐dominated landscapes.

## Summary

- Electrocutions represent a growing threat to nonhuman primate (NHP) populations in urbanized and peri‐urban areas of the Brazilian Cerrado Biome, with black tufted marmosets most frequently impacted.
- This study provides a detailed characterization of gross and microscopic lesions, serving as an essential diagnostic reference for addressing electrocutions within the context of NHP conservation medicine.

## Graphical abstract

Epidemiological and pathological features of electrocutions in free‐ranging new‐world monkeys.

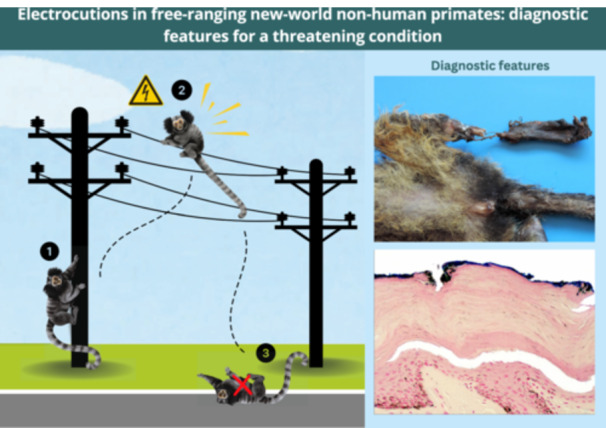

## 1 Introduction

The expansion of agribusiness and other human activities has significantly fragmented the Cerrado Biome (Brazilian savannah), one of Brazil's most biodiverse ecosystems (Pompeu et al. 2024). Habitat loss and environmental changes have driven nonhuman primates (NHPs) into urbanized areas and fragmented habitats (Galán‐Acedo et al. 2019; Alesci et al. 2022; Azofeifa Rojas and Gregory 2022). Among anthropogenic impacts, electric infrastructure networks pose a significant threat to arboreal NHPs due to frequent electrocution incidents resulting in fatalities (Kumar and Kumar 2015; Katsis et al. 2018; Pereira et al. 2020; Cunneyworth and Slade 2021; Sánchez‐Murillo and Arguedas 2021; Aggimarangsee et al. 2022; Linden et al. 2022; Franquesa‐Soler et al. 2023; Yi et al. 2022).

Electrocution is considered a significant cause of injury and mortality in wildlife inhabiting anthropized areas, notably affecting arboreal mammals such as sloths, opossums, and porcupines, as well as birds (Teixeira et al. 2013; Kagan 2016; Almeida et al. 2022). Free‐ranging NHPs are particularly susceptible, with electrocutions documented worldwide (Kumar and Kumar 2015; Katsis et al. 2018; Pereira et al. 2020; Cunneyworth and Slade 2021; Sánchez‐Murillo and Arguedas 2021; Linden et al. 2022; Yi et al. 2022; Franquesa‐Soler et al. 2023; García de la Chica et al. 2023), involving both high‐ and low‐voltage electrical networks (Legendre 1993; Hawkins and Graham 2007; Ozmen and Haligur 2007; Ros et al. 2015; Gal et al. 2016; Nemi et al. 2023).

The vulnerability of NHPs to electrocution in human‐altered environments is heightened by their frequent use electrical infrastructure as travel routes (Kumar and Kumar 2015; Katsis et al. 2018; Pereira et al. 2020; Cunneyworth and Slade 2021; Aggimarangsee et al. 2022; Azofeifa Rojas and Gregory 2022; Linden et al. 2022; Franquesa‐Soler et al. 2023). Electrocution is a leading cause of mortality for some endangered NHP species, yet critical aspects such as the characterization of electrical infrastructure and injury patterns remain poorly understood (Boinski et al. 1998; Al‐Razi et al. 2019; Montilla et al. 2020). These injuries result from the direct passage of electric current through body tissues, often leading to burns, fatal falls, and sudden death (Lee et al. 2000). However, knowledge of electrocution dynamics in NHPs inhabiting urban and peri‐urban environments remains limited (Pereira et al. 2020), particularly regarding identifying and diagnosing electrical injuries, their potential impact on populations, and developing effective mitigation strategies.

Effective conservation of NHPs requires precise diagnostics as a foundation for conservation medicine to address threats like electrocution. In addition, understanding the factors influencing electrocution risk is essential for effective conservation strategies. Seasonal variation may play a role in electrocution rates due to shifts in primate movement patterns, resource availability, and increased human activity in certain periods, potentially affecting exposure to electrical infrastructure (de Andrade 2022; Chaves et al. 2022). Sex and age differences can influence electrocution susceptibility also, as younger and more exploratory individuals may be at higher risk due to inexperience with navigating fragmented landscapes, while sex‐based behavioral differences could also impact exposure (Cunneyworth and Slade 2021). Furthermore, the affected body parts provide key diagnostic insights into electrocution mechanisms, with entry and exit wounds, burns, and secondary trauma patterns helping to distinguish electrocution injuries from other causes of mortality (Mansueto et al. 2021).

Although electrical injuries have been widely reported across various species and populations, detailed gross and microscopic features essential for diagnosing electrocution in NHPs remain poorly documented for many species. This study aimed to demonstrate relevant features of electrocution as a cause of death in free‐ranging NHPs in urban and peri‐urban areas of the Brazilian Cerrado Biome, Federal District, Brazil. Additionally, we explored some epidemiological aspects of the affected populations and provided a comprehensive description of the gross and histopathological features associated with electrocution injuries in NHPs.

## 2 Materials and Methods

### 2.1 Animal Data

We retrieved records of electrocutions in NHPs from the archives of the Veterinary Pathology and Forensics Laboratory (VPFL) at the University of Brasilia, Federal District (FD), Brazil, from January 2019 to December 2022. VPFL serves as a Regional Reference Laboratory under the Brazilian Ministry of Health for diagnosing and surveilling epizootics (outbreaks) in NHPs for the National Control Program for Yellow Fever and other zoonotic infections. In the FD, NHPs are typically found dead or injured by residents, who notify the appropriate authorities (Environmental Military Police and the Local Health Surveillance Service). These authorities collect the animals and direct them for medical treatment or necropsy.

The definitive diagnosis of electrocution was established through a review of clinical histories, gross findings consistent with electrical injuries (including electrical contact points, singed fur, skin erythema and edema, blisters, burns of varying degrees, ulcerated electrothermal injuries, and charred tissues), and characteristic histological alterations (such as epidermal necrosis, smudging of collagen fibers, stretched epidermal nuclei, dermo‐epidermal detachment, a honeycomb‐like epidermal appearance, and skin surface metallization), as previously described (Merck and Miller 2013; Schulze et al. 2016; Sangita et al. 2018; Pereira et al. 2020).

Electrical lesions mapping was conducted based on necropsy records and archived gross images. The classification of NHP electrocution locations (urban, peri‐urban, and natural areas) was determined using geolocation of deaths (when available) and information provided by personnel responsible for carcass collection, according to the geographical and demographic criteria for the Federal District, Brazil (Pereira et al. 2020; IBGE 2022).

Our analysis encompassed data records including the species and genus of NHPs, age categorization (young and adults) (Decanini and Macedo 2008), date of electrocution (month, year, season [Spring, Summer, Fall, and Winter], dry [from May to October] and wet [from November to April] periods), as well as gross and microscopic findings. All collected NHP carcasses were stored under refrigeration (2°–4°C) until necropsies were performed, which occurred within 24 h.

### 2.2 Gross Changes

Electrothermal injuries were classified based on the number of contact points with wires as double, triple, or multiple (four or more contact points). Additionally, the distribution of electrical injuries was assessed concerning the anatomical regions of the head, neck, trunk, limbs, and tail. Specifically, injuries to the limbs were further subdivided into the thoracic limbs (shoulder, arm, forearm, and hand) and pelvic limbs (pelvic region, thigh, leg, and foot), while those to the trunk were divided into the thorax and abdomen.

Electrothermal burns were classified as Grade I, Grade II, Grade III, and Grade IV (Table 1). This classification was adjusted for NHP from the originally adapted for veterinary application in dogs and cats (Merck and Miller 2013). The highest grade of electric burns was assigned when varying grades of injuries were identified within the same animal. In addition to the electrothermal injuries, all gross changes observed in internal organs and tissues were also recorded.

**Table 1:** Classification of electrothermal burns in electrocuted NHPs^a^.

Grade	Criteria
I	Limited to the epidermis, characterized by superficial singeing of fur and skin erythema.
II	Involving the epidermis and partial thickness of the dermis, primarily presenting with blisters, edema, moist lesions, and hyperemia.
III	Affecting all the skin layers, dark brown, often appears with ulcerated lesions exposing subcutaneous tissue and/or superficial muscles.
IV	Extending through all the layers of the skin and underlying tissues, indicating severe injuries with frequent exposure of deep muscles, tendons, bones, or charred tissues.

a Adapted from dogs and cats (Merck and Miller 2013).

### 2.3 Microscopic Evaluation

We also retrieved data from the microscopical evaluation of organs and tissues collected at the gross evaluation and fixed in buffered formalin 10% (pH 7.0). All formalin‐fixed samples were routinely processed, embedded in paraffin, and histological sections stained with hematoxylin and eosin stain (H&E) for microscopic evaluation. Electrothermally injured skin specimens were stained with Perls' Prussian blue stain (PPB) to detect skin metallization formed by tiny iron particles from electrical wiring transferred to the contact points during electrocution (Schulze et al. 2016). We re‐evaluated histological lesions resulting from electrothermal injuries in the skin, including epidermal ulceration, skin metallization (metallization line), crust formation, epidermal hyalinization, stretching of epidermal nuclei, blister formation (honeycomb appearance in some cases), exposure of the basal layer, smudging of collagen fibers, dermal hemorrhage and edema, muscle necrosis and hemorrhage, interstitial muscle edema, congestion, and inflammatory infiltrate. Furthermore, histopathological changes in other organs and tissues were thoroughly examined, with particular emphasis on the brain, heart, and lungs.

### 2.4 Data Analysis and Statistics

We calculated the frequencies (%) for all variables, including NHP species and genus, sex, age categories, and the temporal factors associated with electrocutions, such as month, year, season, and wet or dry periods. Additionally, a descriptive analysis was conducted to determine the frequency distribution of gross pathological findings, microscopic tissue alterations, and the severity of burn injuries.

## 3 Results

### 3.1 Data of Electrocuted NHPs

**Figure 1:**
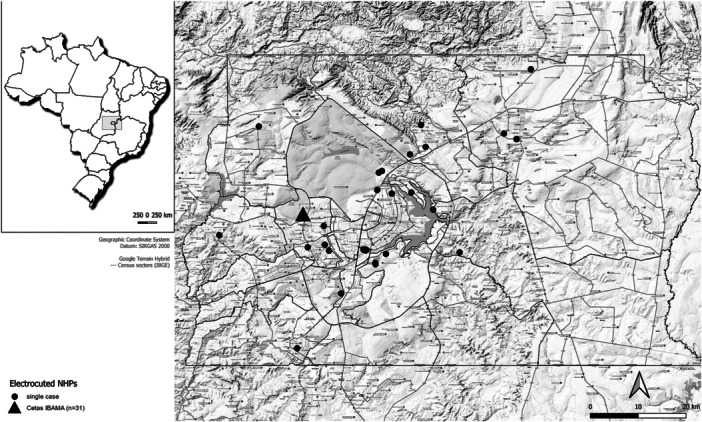
Geographical distribution of electrocutions in nonhuman primates (NHPs) in the Federal District, Brazil, from 2019 to 2022.

Between 2019 and 2022, a total of 429 necropsies were performed on free‐ranging NHPs, with electrocutions accounting for 71 deaths, representing a prevalence of 16.5%. Geolocation data was recorded at the electrocution site for most NHPs (*n *= 40/71) found dead in urban and peri‐urban areas. Additionally, some animals initially rescued alive by Federal District authorities (*n* = 31) were transferred to the Wildlife Rehabilitation Center of the Brazilian Institute of the Environment (CETAS IBAMA) for medical treatment. After their subsequent deaths, necropsies were performed. Due to incomplete geographic data on their original electrocution sites, these individuals were mapped based on the CETAS IBAMA location (Figure 1).

Additionally, some animals initially rescued alive by the authorities of the Federal District (*n* = 31) and included in this study were transferred to the Wildlife Rehabilitation Center of the Brazilian Institute of the Environment (CETAS IBAMA) for clinical care. Following their subsequent deaths, they were taken for necropsies. These individuals were mapped under the CETAS IBAMA location due to incomplete geographic data regarding their original electrocution sites (Figure 1).

**Figure 2:**
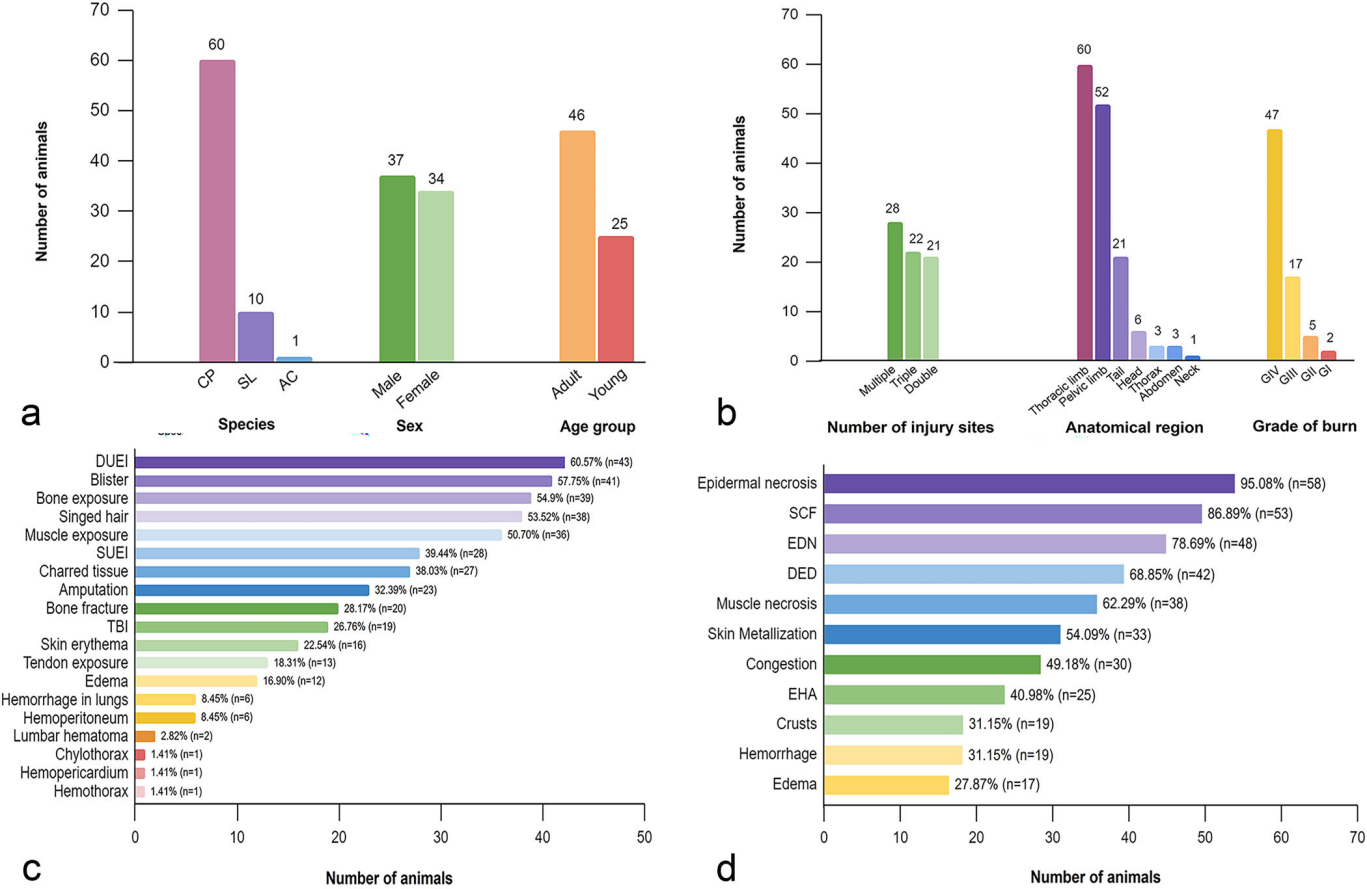
General and pathological data on electrocuted nonhuman primates. (a) Species, sex, and age group of affected animals (*n* = 71). CP *= Callithrix penicillata*. SL = *Sapajus libidinosus*. AC = *Alouatta caraya*. (b) Number of electric injury points, affected anatomical regions, and degree of burns (*n* = 71). (c) Distribution of gross findings (*n* = 71). DUEI = Deep ulcerative electrothermal injury. SUEI = Superficial ulcerative electrothermal injury. TBI = traumatic brain injury. (d) Distribution of microscopic findings in the skin and adjacent muscle tissues (*n* = 61). SCF = smudging of collagen fibers. EDN = epidermal stretched nuclei. DED = dermoepidermal detachment (or blister formation). EHA = epidermal honeycomb aspect.

Black tufted marmosets (*Callithrix penicillata*, 84.5%) were the most frequently affected NHP species by electrocutions in this study, followed by the bearded capuchin monkeys (*Sapajus libidinosus*, 14.1%) and black howler monkeys (*Alouatta caraya*, 1.4%). Most affected NHPs were adults (64.8%) and had a similar number of females (52.1%) and males (47.9%) (Figure 2a). Electrocutions were detected in the Spring (26.8%, *n* = 19), Summer (23.9%, *n* = 17), Fall (21.1%, *n* = 15), and Winter (28.2%, *n* = 20). The number of electrocutions was higher in the rainy season (59.1%, *n* = 42) than in the dry season (40.9%, *n* = 29).

### 3.2 Gross Findings

**Figure 3:**
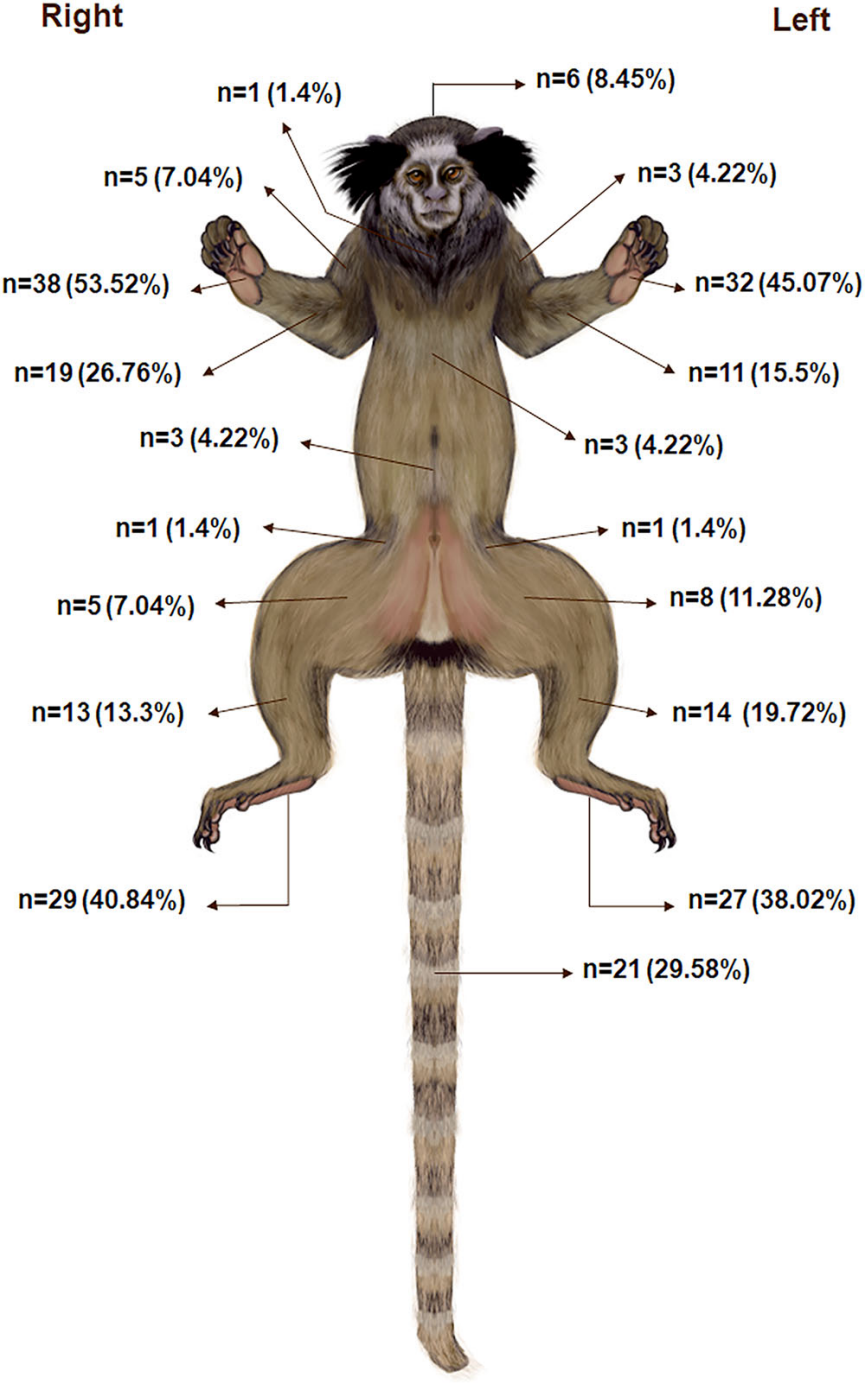
Anatomical distribution of regions affected by electrocution injuries in nonhuman primates.

Double, triple, or multiple injury sites resulting from contact with electric wires were identified, revealing no differences between their frequencies (Figure 2b). Concerning the anatomic sites of electrical injuries, the limbs were most affected, including the forelimbs (84.5%) and hind limbs (73.2%), followed by the tail (29.5%), abdomen (4.3%), thorax (4.3%), head (4.3%), and neck (1.4%) (Figure 2b). The distribution of electrocution injuries across anatomical locations of the body is illustrated in Figure 3.

**Figure 4:**
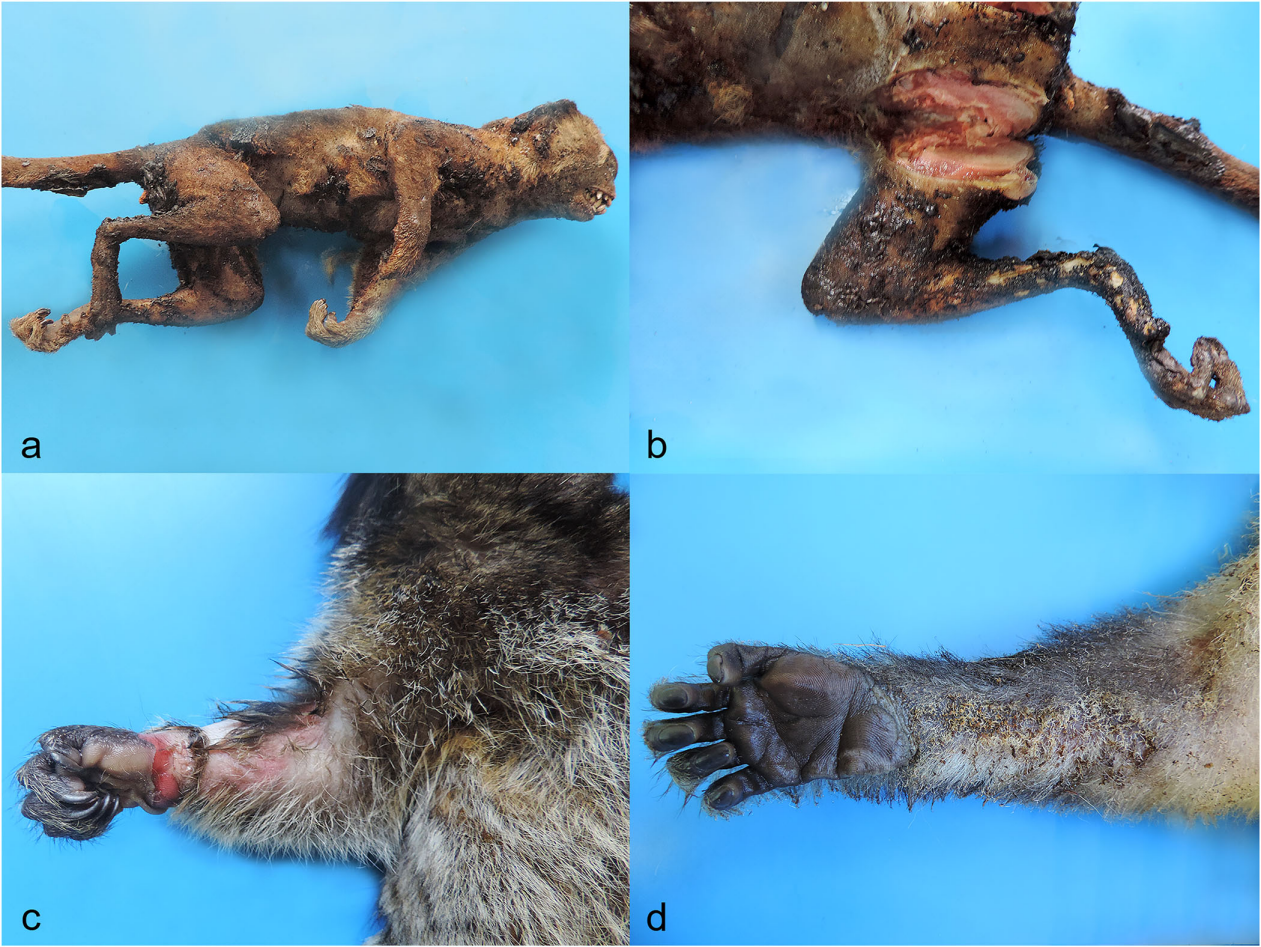
Gross injuries in the skin of electrocuted marmosets. Grade of burns. (a) and (b) Grade IV burns characterized by blackned and charred tissues and burns affecting deep muscle tissues. (c) Grade III burn evidencing ulceration, exposure of muscle tissues and erythema adjacent. (d) Grade II burn showing scorched furs, edema, and small multifocal irregular blister areas (gray discoloration) in the palmar surface.

Grossly, all electrocution‐associated injuries recorded in NHPs, from the most to the least frequent, are presented in Figure 2c. Hands were the most injured anatomic region (45.0%–53.5%) in electrocuted animals, followed by the forearms (15.5%–26.7%) and arms (4.2%–7.0%) in the thoracic limbs. In the pelvic limbs, the feet (38.0%– 40.8%) exhibited the highest frequency of injuries, followed by the leg (13.3%–19.7%), thigh (7.0%–11.3%), and pelvic region (1.4%) (Figure 3). Most of the electrical burns (Figure 2b) were categorized as Grade IV (66.2%) (Figure 4a and Figure 4b), followed by Grade III (23.9%) (Figure 4c), Grade II (7.0%) (Figure 4d), and Grade I (2.8%).

**Figure 5:**
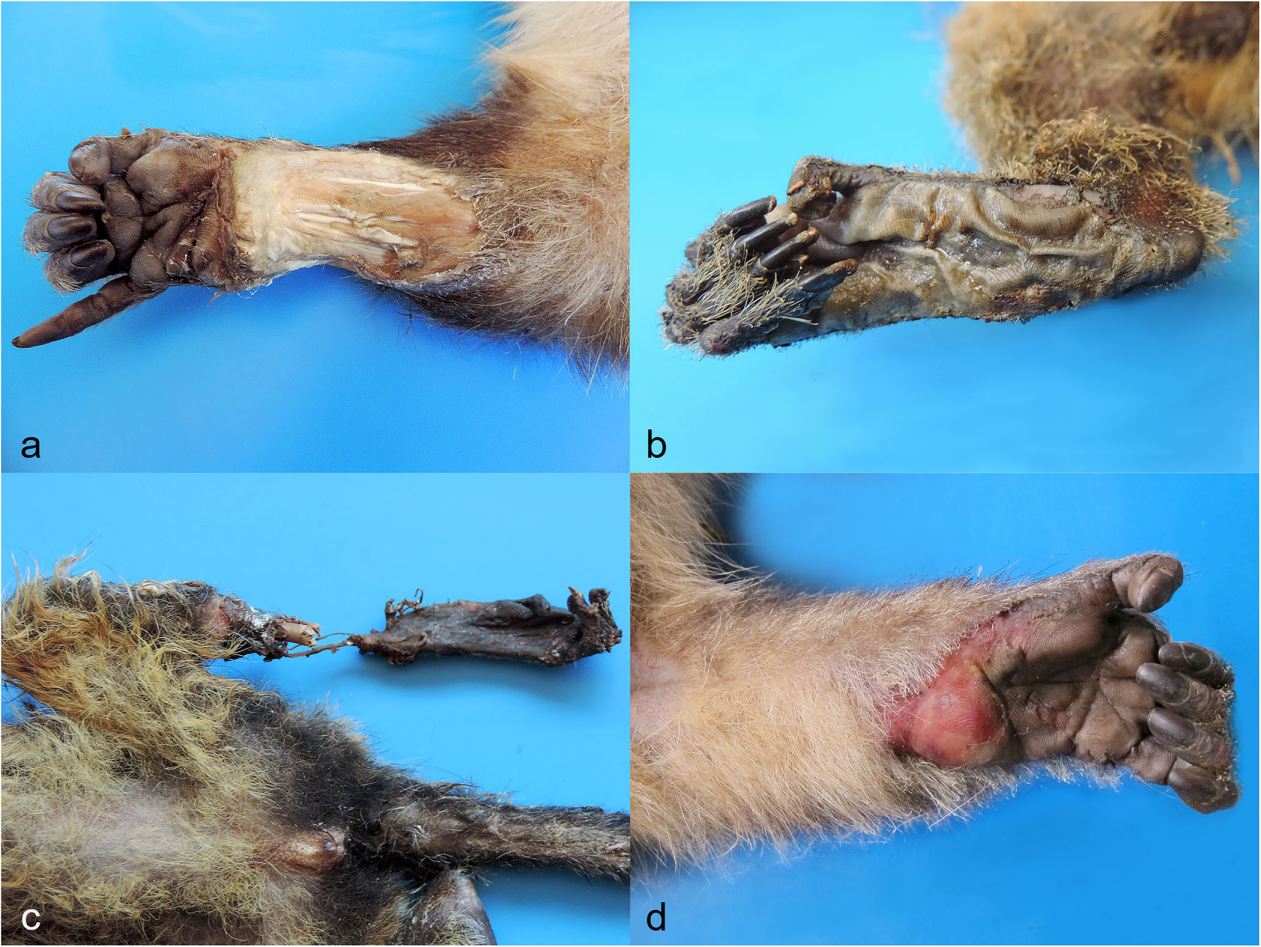
Gross injuries in electrocuted NHPs. (a) Black tufted marmoset, right upper limb. Deep ulcerative electrothermal injury with muscle and tendon exposure. (b) Black tufted marmoset, right pelvic limb. Blisters and singed hair. (c) Black tufted marmoset, left pelvic limb. Charred tissues, bone fracture and exposure, and amputation. (d) Bearded capuchin monkey, left upper limb. Skin erythema and swelling.

Deep ulcerative electrothermal injuries, muscle and tendon exposure (Figure 5a), singed hair and blisters (Figure 5b), bone exposure and fractures, limb amputations, superficial ulcerative electrothermal injuries, and charred tissues (Figure 5c) were the most commonly observed gross lesions in electrocuted NHPs (Figure 2c). Additionally, traumatic brain injuries, skin erythema and edema (Figure 5d), pulmonary hemorrhage, hemoperitoneum, lumbar hematoma, chylothorax, hemopericardium, and hemothorax were also documented in electrocuted NHPs (Figure 2c). Bone exposure (*n* = 39/71, 54.9%), limb amputation (*n* = 23/71, 32.4%), and bone fractures (*n* = 20/71, 28.1%) were the most frequent skeletal injuries associated with electrocution in NHPs.

### 3.3 Microscopic Changes

**Figure 6:**
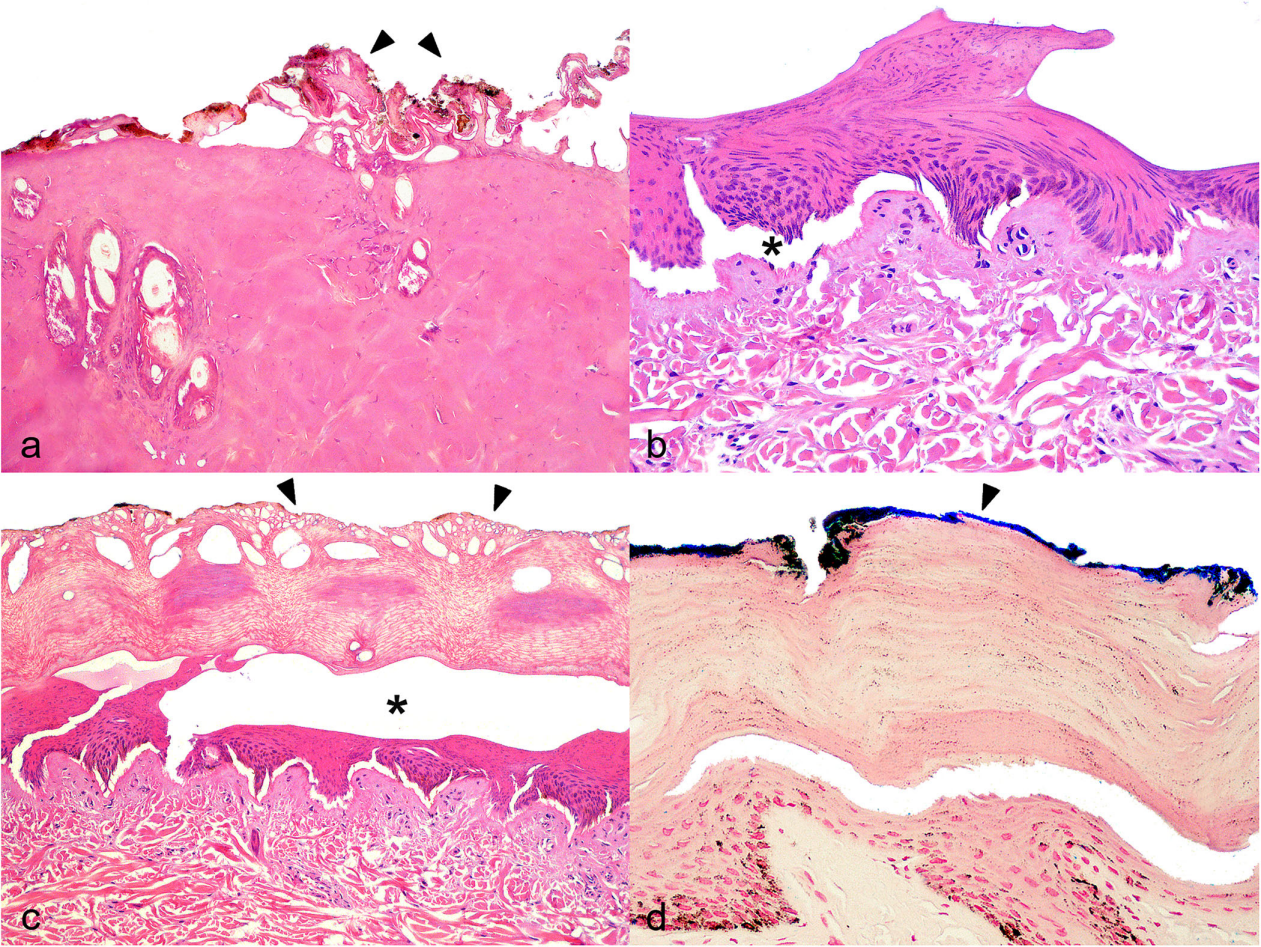
Histological findings in the skin of electrocuted NHPs. (a) Epidermal necrosis (arrowhead) and marked smudging of collagen fiber in the dermis (H&E, obj. 10x). (b) Epidermal stretched nuclei and dermoepidermal detachment (asterisk) (H&E, obj. 20x). (c) Epidermal honeycomb aspect (arrowheads) and subcorneal blister formation (asterisk) (H&E, obj. 10x). (d) Metallization of the epidermis (arrowhead) (PPB stain, obj. 20x).

Microscopic findings identified in the skin of electrocuted NHPs are shown in Figure 2d. Epidermal necrosis (Figure 6a), smudging of collagen fibers (Figure 6a), stretched epidermal nuclei (Figure 6b), dermo‐epidermal detachment (Figure 6b) (or blister formation) (Figure 6c), epidermal honeycomb aspect (Figure 6c), muscle necrosis, and metallization of the skin surface (Figure 6d) were the most frequent electrothermal injuries observed in the skin of affected NHPs. Additionally, congestion, an epidermal honeycomb appearance, crust formation, hemorrhage, and edema were also noted. The main histological findings detected in the lungs, heart, and brain of NHPs affected by electrocution were congestion, hemorrhage, and edema (Table 2). Microscopic changes in other organs and tissues were unremarkable.

**Table 2:** Histological findings in some internal organs of electrocuted NHPs.

Findings	Organ	n	%
Congestion	Lung	70	98.59
	Heart	41	57.75
	Brain	45	63.39
Hemorrhage	Lung	45	63.39
	Heart	45	63.39
	Brain	8	11.27
Edema	Lung	32	45.07

## 4 Discussion

Human‐induced alterations in natural environments, particularly urbanized areas, can profoundly impact wildlife and pose significant threats to their survival (Teixeira et al. 2013; Kagan 2016; Almeida et al. 2022; Chaves et al. 2022). This is exemplified by the electrocution of free‐ranging NHPs in urban and peri‐urban areas of the Brazilian Cerrado Biome. Accurately identifying the primary causes of mortality, such as electrocution, and conducting thorough diagnostic assessments of affected individuals are critical for conservation efforts.

Our findings revealed that electrocutions accounted for 16.5% of NHPs deaths in the Federal District (FD). Black tufted marmosets (*Callithrix penicillata*) were predominantly affected, as previously observed in Midwestern Brazil (Pereira et al. 2020). These small arboreal primates, native to the Brazilian Savannah Biome (Cerrado), have adapted well to anthropized environments (Goulart et al. 2010; Pereira et al. 2020). Their arboreal habits lead them to access uninsulated electrical wires with their hands while moving through trees (Dittus 2020), which significantly increases their vulnerability to electrocutions in densely human‐populated areas.

In contrast, howler and capuchin monkeys, which inhabit more undisturbed forests (Cunha et al. 2019; Falótico et al. 2021), were less represented in this study. The higher prevalence of electrocution cases among NHPs (16.5%) compared to a previous study focused solely on marmosets (11.0%) (Pereira et al. 2020) suggests a possible increase in incidents, potentially driven by habitat loss resulting from urban and agribusiness expansion (Galán‐Acedo et al. 2019; Alesci et al. 2022; Azofeifa Rojas and Gregory 2022; Pompeu et al. 2024).

No sex bias was observed in our study, though adults were most frequently electrocuted, consistent with findings in Midwestern Brazil (Pereira et al. 2020). In contrast, juvenile rhesus monkeys were more vulnerable in India (Kumar and Kumar 2015), which may be related to their lack of experience in traversing fragmented environments. Sex‐based behavioral differences could also impact exposure to electrical infrastructure (Cunneyworth and Slade 2021), while, the smaller size of juvenile marmosets, may lower their risk by limiting contact with multiple electrical wires (Pereira et al. 2020).

The higher incidence of electrocutions during the rainy season in this study align with previous findings in free‐ranging rhesus monkeys (Kumar and Kumar 2015) and can be attributed to multiple factors. Rain significantly reduces electrical resistance in both animal skin and wiring (Koumbourlis 2002), enhancing conductivity and electrocution risks. Additionally, seasonal changes in movement patterns and resource availability (de Andrade 2022; Chaves et al. 2022) likely influence NHP exposure to electrical infrastructure during wet periods. While specific skin electric resistance data for NHPs is unavailable, similar patterns observed in human and bovine tissues suggest moisture is a key contributor to increased electrocution rates (Schulze et al. 2016).

Our analysis revealed distinctive injury patterns associated with electrocution. Limbs (85.83%) and tail (8.75%) were the primary sites of electrical injuries, with hands more frequently affected than feet detected in NHPs. This likely reflects how NHPs initially contact electrical wires when moving through trees (Kumar and Kumar 2015; Pereira et al. 2020).

The high prevalence of multiple contact points (39.5%) and severe burns can be explained by several mechanisms: tetanic muscle contractions induced by electrical currents force animals to grip power lines, prolonging exposure (Spies and Trohman 2006; Mittal and Bohnert 2023); electrical energy converts to heat at contact points, generating tissue fluid vaporization and carbonization (Hunt et al. 1976; Viner 2018); and the high‐voltage electrical networks (1,000‐volt) with narrow spacing between uncoated wires (20 cm) in the region exacerbate injury severity, especially in smaller‐bodied species like marmosets.

Grade IV (66.2%) and Grade III (23.9%) burns were the most common injury patterns, indicating severe thermal tissue damage resulting from electrical energy conversion into heat (Schulze et al. 2016; Mansueto et al. 2021). The resistance of the epidermal keratin layer at electrical contact points generates heat, tissue fluid vaporization, and carbonization (Hunt et al. 1976; Spies and Trohman 2006; Viner 2018), further contributing to the severe burns and high mortality recorded in this study.

Secondary trauma from falls also contributed to injuries, including bone fractures, traumatic brain injuries, amputations, and hemorrhages, similar to findings in electrocuted rhesus monkeys and raptors (Kumar and Kumar 2015; Kagan 2016). These injuries may result from violent muscle contractions during electrocution causing fractures, spinal injuries, and joint dislocations (Spies and Trohman 2006), while hemorrhages may result from vascular tears or muscle contractions (Kagan 2016). The case of chylothorax in an electrocuted marmoset suggests thoracic duct injury, consistent with previous findings (Barros et al. 2024).

Nonfatal electrocutions can significantly impact NHP populations, as individuals with amputated limbs may struggle to survive. In our study, over 32% of examined cases showed member amputations, indicating that the number of affected NHPs with sequelae in FD may be higher. Supporting this finding, a study with electrocuted rhesus macaques found that 68% of these animals survived but suffered lasting impairments (Kumar and Kumar 2015).

Microscopic examination showed characteristic skin electrothermal injuries in the skin of affected NHPs, including epidermal necrosis, smudged collagen fibers, stretched epidermal nuclei, and dermo‐epidermal detachment. These findings arise from Joule heating (Al‐Alousi 1990; Viner 2018), collagen denaturation, and dermal edema (Nikolenko and Mitin 1986; Aquila et al. 2018).

Blister formation due to dermo‐epidermal separation exhibited a honeycomb pattern in about 41% of cases, resulting from desmosomal disruption due to thermal injury (Üzün et al. 2008; Mondello et al. 2018; Schulze et al. 2016) and tissue fluid evaporation (Sangita et al. 2018). Skin metallization, observed in over 50% of cases, occurs when microscopic metal particles from wiring deposit onto the skin (Marcinkowski and Pankowski 1980; Schulze et al. 2016), with particles varying by conductor composition (Boracchi et al. 2019).

Other common microscopic lesions included organ congestion (particularly in the brain, heart, and lungs), pulmonary edema, and hemorrhages, consistent with findings in electrocuted in rhesus macaques (Kumar and Kumar 2015) and marmosets (Pereira et al. 2020). While pulmonary congestion may be a nonspecific finding (Gal and Castillo‐Alcala 2022), lung edema likely results from altered vascular permeability and hydrostatic pressure (Unger and Martin 2023).

Myocardial and pulmonary hemorrhages affected over 60% of affected NHPs in our study, aligning with previous reports (Kumar and Kumar 2015; Pereira et al. 2020). Petechiae in multiple organs, linked to increased blood pressure and venous congestion following cardiac arrest (Karger et al. 2002), were frequently noted, as were myocardial hemorrhages, a hallmark of human electrocution (Shetty et al. 2014; Favia et al. 2021).

Our findings raise concerns about the impact of electrocutions on free‐ranging NHP populations in urban and peri‐urban areas of the Federal District. Despite knowledge gaps regarding NHP population densities, home range dynamics, movement patterns, and habitat use in the study region, our results suggest that implementing preventive measures is urgently needed. Redesigning electrical wiring, such as implementing underground, insulated, or coated cables, could be an initial step to mitigate risks, enhance conservation efforts, and promote coexistence in anthropogenic landscapes. Additional research on population dynamics and distribution in the Federal District is essential for developing more comprehensive strategies to prevent electrocutions and other life‐threatening hazards for free‐ranging NHP populations.

## Author Contributions

**Rafaela M Barros:** conceptualization (equal), data curation (equal), formal analysis (equal), investigation (equal), methodology (equal), writing – original draft (equal), writing – review and editing (equal). **Isabel L Macêdo:** conceptualization (equal), formal analysis (equal), investigation (equal), methodology (equal), writing – original draft (equal), writing – review and editing (equal). **Davi E R Sousa:** conceptualization (equal), investigation (equal), methodology (equal), writing – original draft (equal), writing – review and editing (equal). **Liz A Cerqueira:** conceptualization (equal), investigation (equal), methodology (equal). **Yasmin N G Fonseca:** conceptualization (equal), investigation (equal), methodology (equal). **Ana L V Sousa:** conceptualization (equal), investigation (equal), methodology (equal). **Antonio D Santos:** methodology (equal), validation (equal). **Cristiano B Melo:** conceptualization (equal), writing – original draft (equal), writing – review and editing (equal). **Márcio B Castro:** conceptualization (lead), investigation (equal), methodology (equal), writing – original draft (lead), writing – review and editing (lead).

## Ethics Statement

As an Official Regional Laboratory for the Brazilian Ministry of Health for the diagnosis of Yellow Fever and other epidemic diseases in NHPs, the Laboratory of Veterinary Pathology and Forensics (LVPF‐UnB) routinely receives dead free‐ranging NHPs for post‐mortem examination and Yellow Fever testing. These animals are collected by State Health Surveillance Services as part of the Brazilian National Program for Yellow Fever Control. This study utilized data and samples from the necropsy records archived at the LVPF‐UnB. Therefore, ethical approval is not required for this study.

## Conflicts of Interest

The authors declare no conflicts of interest.

## Data Availability

The authors have nothing to report.
